# Supplementary material for: Splice-Junction-Based Mapping of Alternative Isoforms in the Human Proteome
Source: Cell Rep. Author manuscript; Available in PMC 2020 Jan 15. (PMC6961840; doi:10.1016/j.celrep.2019.11.026)
Supplement: 2 [file NIHMS1546469-supplement-2.zip › DF1/PXD006675/VentricularSeptum/VentricularSeptum_3_TTN_SNCTVSVHVSEPPR.pdf]

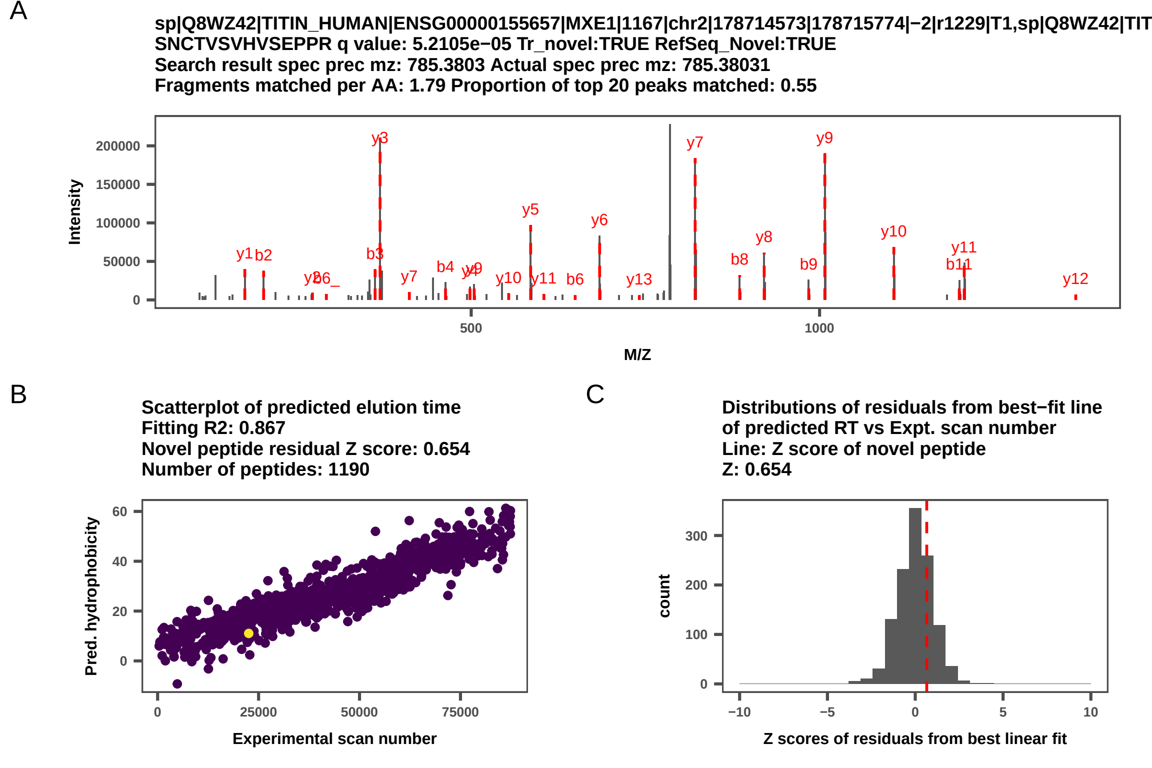
A
sp|Q8WZ42|TITIN_HUMAN|ENSG00000155657|MXE1|1167|chr2|178714573|178715774|−2|r1229|T1,sp|Q8WZ42|TIT
SNCTVSVHVSEPPR q value: 5.2105e−05 Tr_novel:TRUE RefSeq_Novel:TRUE
Search result spec prec mz: 785.3803 Actual spec prec mz: 785.38031
Fragments matched per AA: 1.79 Proportion of top 20 peaks matched: 0.55
Intensity
200000
150000
100000
50000
0
y1
b2
y3
b3
y7
b4
y9
y10
y5
y11
b6
y6
y13
y7
b8
y8
b9
y9
y10
y11
b11
y12
500
1000
M/Z
B
Scatterplot of predicted elution time
Fitting R2: 0.867
Novel peptide residual Z score: 0.654
Number of peptides: 1190
Pred. hydrophobicity
60
40
20
0
0
25000
50000
75000
Experimental scan number
C
Distributions of residuals from best−fit line
of predicted RT vs Expt. scan number
Line: Z score of novel peptide
Z: 0.654
count
300
200
100
0
−10
−5
0
5
10
Z scores of residuals from best linear fit
